# Five-year response to growth hormone in children with Noonan syndrome and growth hormone deficiency

**DOI:** 10.1186/s13052-015-0183-x

**Published:** 2015-10-06

**Authors:** Niki Zavras, Cristina Meazza, Alba Pilotta, Chiara Gertosio, Sara Pagani, Carmine Tinelli, Mauro Bozzola

**Affiliations:** Fondazione IRCCS San Matteo, Pavia, Italy; Internal Medicine and Therapeutics Department, University of Pavia, Auxology Research Centre, Fondazione IRCCS Policlinico San Matteo, Piazzale Golgi, 2 27100 Pavia, Italy; Auxoendocrinology Center, Pediatric Department, University of Brescia, Spedali Civili, Brescia, Italy; Clinical Epidemiology and Biometric Unit, Fondazione IRCCS San Matteo, Pavia, Italy

**Keywords:** Noonan syndrome, Children, Recombinant human growth hormone therapy, Short stature

## Abstract

**Background:**

Noonan syndrome (NS) is an autosomal dominant disorder characterized by specific features including short stature, distinctive facial dysmorphic features, congenital heart defects, hypertrophic cardiomyopathy, skeletal anomalies and webbing of the neck. Molecular screening has shown that the majority of individuals with NS have a mutation in the PTPN11 gene. Noonan syndrome children may show an impaired growth hormone (GH)/insulin-like growth factor axis. Moreover, recombinant human GH (rhGH) has been shown to improve growth rate in patients with NS, although data are still limited.

**Methods:**

In the present study, we assessed growth response following GH therapy (0.25 mg/Kg/week) in 5 (2 M and 3 F) GH-deficient NS patients (NSGHD, mean age 8.5 years) and in 5 (2 M and 3 F) idiopathic GH deficient (IGHD, mean age 8.6 years) patients. We also evaluated the safety of rhGH therapy in NS patients with GHD.

**Results:**

At the beginning of GH treatment, height and growth rate were statistically lower in NSGHD children than in IGHD ones. During the first three years of rhGH therapy, NSGHD patients showed a slight improvement in height (from −2.71 SDS to −2.44 SDS) and growth rate (from −2.42 SDS to −0.23 SDS), although the values were always significantly lower than in IGHD children. After five years of rhGH treatment, height gain was higher in IGHD children (mean 28.3 cm) than in NSGHD patients (mean 23.6 cm).

During the first five years of rhGH therapy, regular cardiological and haematological check-ups were performed, leading to the conclusion that rhGH therapy was safe.

**Conclusions:**

In conclusion, pre-pubertal NS children with GHD slightly increased their height and growth rate during the first years of GH therapy, although the response to rhGH treatment was significantly lower than IGHD children. Furthermore, the therapy appeared to be safe since no severe adverse effects were reported, at least during the first five years. However, a close follow-up of these patients is mandatory, especially to monitor cardiac function.

## Introduction

Noonan syndrome (NS) is a disorder first reported by the paediatric cardiologist Jaqueline Noonan in 1963 [1] and described in detail a few years later by the same author [2] and then by other groups [3]. It is an autosomal dominant disorder, affecting 1 in 1,000–2,500 live births with no sex predominance, and is the most common syndromal cause of congenital heart disease, except for Down’s syndrome.

Noonan syndrome is characterized by short stature, distinctive facial dysmorphic features including hypertelorism, down-slanting palpebral fissures and low-set posteriorly rotated ears, congenital heart defects, hypertrophic cardiomyopathy, skeletal anomalies and webbing of the neck. Other relative common features are bleeding diathesis, ectodermal anomalies, lymphatic dysplasia, cryptorchidism and cognitive deficits [4–7]. The clinical features become more evident with age [5] and the diagnosis is principally clinical. At present, genetic testing is available to confirm or make the diagnosis. In fact, NS is caused by mutations in genes that encode proteins of the RAS-MAPK signal transduction pathway [8]. Molecular screening has shown that the majority of individuals with a diagnosis of NS have a mutation in the PTPN11 gene (30–60 %) that encodes for the protein tyrosine phosphatase SHP2 [8]. Other mutations have been described in KRAS, RAF1, SOS1, NRAS and SHOC2 genes. However, a failure to identify a mutation does not exclude the diagnosis of NS [9, 10].

Growth in NS has been a subject of interest since its first description in 1963 [1]. Short stature is reported in 50–70 % of patients with NS and the majority of affected patients have height below the third percentile [2–7, 11, 12]. Mean adult stature ranges from 145–162.5 cm for men and 135–151 cm for women [11]. Birth weight and length are typically normal, but subsequent delayed growth rate affects height, weight and bone development. After the first months of life there is a progressive deceleration of linear growth. Bone age after the age of 5 years is generally retarded (about 2 years). Puberty is often delayed and catch-up growth does not occur.

Children with NS are not usually growth hormone deficient (GHD), but may show some abnormalities in the GH/insulin-like growth factor (IGF) axis [13, 14]. Recombinant human GH (rhGH) has been shown to improve growth rate in NS patients as in patients with Turner’s syndrome [15, 16], but data are yet still limited. Most of the evidence of rhGH effects on NS children growth comes from observational studies in small numbers of patients and without randomization or control groups (Table 1).

**Table 1:** Published data from studies of GH treatment in Noonan patients

Reference	No. patients (M/F)	Height SDS at start of GH treatment^#^	GH dose (mg/kg/week)	Duration of GH treatment (yrs)	Height SDS at last observation^#^
Ahmed et al. 1991 [25]	6 (3/3)	From −3.5 to −2.3	0.18	1	-
Thomas et al. 1993 [31]	5 (4/1)	From −4.2 to −2.2	0.35	2.9	From −3.3 to −1.6
Municchi et al. 1995 [32]	4 (0/1)	From −1.9 to 0.2*	0.17	3	From −0.9 to 0.9*
Cotterill et al. 1996 [33]	30 (19/11)	−3.01 ± 0.1	0.33	1	−2.36 ± 0.1
de Schepper et al. 1997 [15]	23 (18/5)	−2.28 ± 0.68	0.35	1	−1.78 ± 0.76
Soliman et al. 1998 [34]	12 (3/9)	−2.2 ± 0.6	0.28	1	1.45 ± 0.3
MacFarlane et al. 2001 [35]	23 (16/7)	−2.7 ± 0.4	0.33	3	−1.9 ± 0.9
Ogawa et al. 2004 [36]	15 (8/6)	−2.8 ± 0.7	0.17	2	−2.2 ± 0.5
Ferreira et al. 2005 [22]	14 (10/4)	−3.5 ± 1.0 (PTPN11 mutation)	0.29	3	0.76 ± 0.41 (PTPN11 mutation)
		−3.4 ± 1.0 (no PTPN11 mutation)			1.74 ± 0.10 (no PTPN11 mutation)
Binder et al. 2005 [37]	29 (19/10)	−3.5 ± 1.0 (PTPN11 mutation)	0.30	1	0.66 ± 0.21 (PTPN11 mutation)^$^
		−3.4 ± 1.0 (no PTPN11 mutation)			1.26 ± 0.36 (no PTPN11 mutation)^$^
Osio et al. 2005 [38]	25 (12/13)	−2.9 ± 0.4	0.23-0.46	1-9	−1.2 ± 1.0
Limal et al. 2006 [23]	35 (19/16)	−3.1 ± 0.9 (PTPN11 mutation)	0.30-0.46	2	−3.1 ± 1.4 (PTPN11 mutation)
		−2.4 ± 0.8 (no PTPN11 mutation)			−2.0 ± 0.9 (no PTPN11 mutation)
Noordam et al. 2008 [21]	29 (21/8)	From −4.1 to −1.8	0.35	3-10.3	From −3.0 to −0.3
Choi et al. 2012 [24]	28 (14/4)	−2.8 ± 0.9	0.46	1	−2.0 ± 0.9

^#^Mean ± standard deviation or range

*Noonan reference [39]

^$^Change in height SDS

In the present study, we assessed growth response following GH therapy in GH-deficient NS patients (NSGHD) and compared it with idiopathic GH deficient (IGHD) sex and age-matched patients. We also evaluated the safety of rhGH therapy in NS patients with GHD.

## Patients and methods

### Patients

We studied 10 pre-pubertal children, 5 NS patients (2 males and 3 females) with GHD and 5 patients (2 males and 3 females) with IGHD. The diagnosis of GHD was established when GH response to at least two pharmacological stimuli was lower than 10 ng/ml in the presence of short stature, reduced growth velocity and delayed bone age [17]. None of the patients had diabetes insipidus, chromosomal abnormalities, dysmorphic syndromes or acquired GHD, after careful clinical evaluation. All subjects showed normal thyroid and adrenal function. Magnetic resonance imaging of the hypothalamus and pituitary region was performed in all patients.

In NS children, the mean chronological age was 8.5 years (standard deviation, SD: 3.1), height was −2.70 standard deviation score (SDS) (SD: 0.42), growth velocity was −2.24 SDS (SD: 3.80) and body mass index (BMI) was −1.54 SDS (SD: 0.31). In patients with IGHD, chronological age was 8.6 years (SD: 4.0), height was −1.82 SDS (SD: 0.71), growth velocity was −1.08 SDS (SD: 1.44) and BMI was −0.99 SDS (SD: 0.82).

The diagnosis of NS was clinically established by physicians experienced with NS on the basis of the Van der Burgt criteria [6, 7]. Genetic analysis was also performed and showed different mutations. In 4 out of 5 patients we found four different PTPN11 mutations affecting different exons of the gene. The other patient showed a mutation in the KRAS gene. Every NS child had a proper cardiological, endocrinological (thyroid and adrenal) and oral glucose tolerance test evaluation, before starting rhGH therapy. In our NS patients no major cardiological criteria were detected.

All IGHD children showed normal birth weight and length for gestational age. Anthropometric data were measured by experienced physicians both at the time of GHD diagnosis and at regular follow-up intervals using the Tanner standard [18].

According to the recommendations for the use of rhGH in children, rhGH therapy was administered at the weekly dose of 0.25 mg/kg subcutaneously subdivided in 6 daily doses in the evening in both the NSGHD and IGHD children.

### Ethics, consent and permissions

Informed consent regarding diagnostic and therapeutic approaches was obtained from parents of all subjects.

### Statistical analysis

The results for quantitative variables are expressed as mean values and SD (the Shapiro-Wilk test was used to test the normal distribution of variables). Linear regression models for repeated measures were used to analyse differences over time and among different patient groups. Two-sided *p* values <0.05 were considered statistically significant. Stata 13.0 (StataCorp 2013, College Station, Texas) was used for all computations.

## Results

Children affected by NS and GHD and children with only GHD had comparable chronological age and BMI at the time of endocrinological evaluation. On the contrary, they differed in height and growth rate, which were statistically lower in NSGHD children than in IGHD ones.

**Fig. 1:**
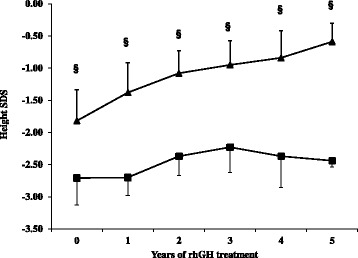
Height before and during the first five years of rhGH replacement therapy in NSGHD (closed square) and IGHD patients (closed triangle). Data are represented as mean and SD. ^§^ *p* < 0.005 NSGHD versus IGHD at the corresponding time. *p < 0.05 in NSGHD children versus time = 0

**Fig. 2:**
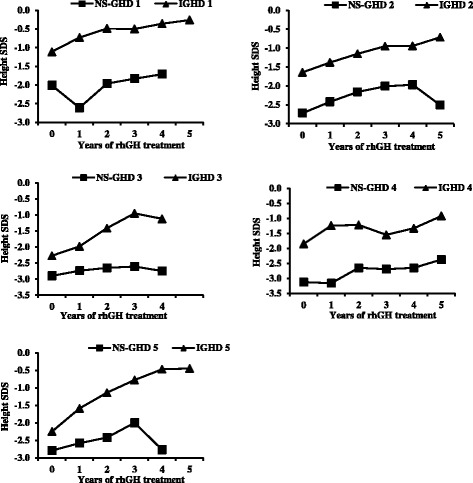
Height patterns of each NSGHD child paired with a sex and age-matched IGHD patient

During the years of therapy, NSGHD patients showed a slight improvement in height, from a mean value of −2.71 SDS to −2.44 SDS (Fig. 1). This increase was evident during the first three years of treatment, with a statistical significant increase in height during the second (*p* = 0.026) and third year (*p* = 0.022) of treatment compared to basal values; in the last two years height SDS values appeared to be constant. On the contrary, IGHD patients showed a good response to rhGH therapy during the follow-up, with a statistically significant increase from −1.82 SDS to −0.59 SDS. Therefore, the patterns of height SDS values were significantly different between the two groups during the first five years of rhGH treatment. The different height patterns of each NSGHD child paired with a sex and age-matched IGHD control are shown in the panels of Fig. 2. Each NSGHD patient showed a significantly lower height than the corresponding sex and age-matched IGHD one.

The height gain after five years of rhGH treatment was higher in IGHD children (28.3 cm, SD: 4.7; 1.13 SDS, SD: 0.39) than in NSGHD patients (23.6 cm, SD: 2.2; 0.29 SDS, SD: 0.28), although the difference was not statistically significant (*p* = 0.061).

**Fig. 3:**
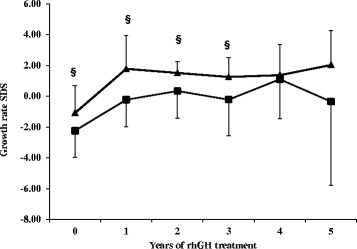
Growth velocity before and during the first five years of rhGH replacement therapy in NSGHD and IGHD patients. Data are represented as mean and SD. ^§^ *p* < 0.005 NSGHD versus IGHD at the corresponding time. *p < 0.05 in NSGHD children versus basal time = 0

Growth rate was also different between the two groups at baseline and during the five-year follow-up. This difference is not due to the pubertal development of patients, since during the five years of follow-up four patients out of five entered puberty in both groups. During the first year of therapy, growth rate increased in a similar way in NSGHD and IGHD subjects (from a mean value of −2.42 SDS to −0.23 SDS and from −1.09 SDS to 1.78 SDS, respectively) (Fig. 3). In particular, in NSGHD patients growth rate significantly increased during the second year of treatment (*p* = 0.032). Then, growth rate values remained quite constant in both groups, being always higher in IGHD children.

Body mass index values did not significantly change during the first five years of rhGH therapy in both groups of subjects, although the values were always higher in IGHD than in NSGHD children (data not shown).

During the first five years of rhGH therapy, regular cardiological and haematological check-ups were performed; the results suggest that rhGH therapy is safe in this kind of patient.

## Discussion

In this study we evaluated the growth-promoting efficacy of rhGH therapy in children with NSGHD comparing them with idiopathic GHD children only, during the first five years of therapy. Both groups of patients were treated with the dose of GH usually used for IGHD children. It is interesting to underline that this dose is lower than the dose used by many authors to treat NS patients without GHD.

Short stature is a typical feature of children with NS. The cause of short stature is the affection of proteins of the RAS-MAPK pathway. Furthermore, a genotype-phenotype correlation has also been hypothesized. In fact, short stature is most prevalent in patients with mutations in the PTPN11 gene, probably due to the fact that SHP2 is involved in GH receptor signalling.

Since 2007, the use of rhGH in patients with NS has been approved by the U.S. Food and Drug Administration (FDA) at a high dose (33–66 μg/kg/day) [19]. The reported studies (Table 1), despite the small sample size of cohorts and the short time of treatment, indicate that short-term rhGH therapy increases mean height SDS in short children with Noonan syndrome and the height gain was reported to be 0.6-2.0 SDS, which is equivalent to 4–13 cm [20]. Noordam et al. published data which concluded that rhGH improves adult height (mean gain in height-SDS of +1.3) in Noonan syndrome with and without the PTPN11 mutation [21]. In some studies, gene mutations in Noonan patients seem also to influence the response to GH therapy. In fact, decreased IGF-I levels and reduced response to GH treatment in NS children with PTPN11 mutations have been reported, suggesting a mild form of GH resistance [22, 23]. On the contrary, in other studies no significant differences in height SDS, height velocity and serum IGF-I level in response to rhGH treatment were found between children with or without PTPN11 mutations [21, 24].

Unfortunately, in our present study we were not able to conclude whether PTPN11 mutations could affect height or response to rhGH therapy, since four out of five patients present mutations in this gene.

In Italy, treating Noonan children with rhGH is not allowed unless a condition of GH deficiency is found, as in our patients. However, disturbances in GH secretion (low nocturnal levels of GH or an unusual pulsatility of GH levels) or action seem to be involved in the pathogenesis of NS [25, 26].

Our NSGHD patients showed only a slight increase in height and growth rate during rhGH therapy and this increase was more evident during the second and third year of treatment. The response was significantly lower than that shown by IGHD only age- and sex-matched children. Furthermore, we administered the same doses of GH that are used in idiopathic GHD children, which are lower than those used in other studies with Noonan GH sufficient children. This different response to GH therapy is not due to the pubertal development of patients, since during the five years of follow-up four out of five patients entered puberty in both groups. However, since puberty in NS children is typically delayed, early initiation of rhGH therapy and long pre-pubertal duration should result in improved height at pubertal onset and in a less affected final height [27].

According to other studies, we found that BMI is not affected by rhGH treatment. However, it has been shown that favourable changes in fat mass and body composition are achievable during rhGH treatment in NS patients [28].

Noonan syndrome is generally associated with cardiac defects, such as mild or severe hypertrophic cardiomiopathy, which has raised concerns related to the anabolic effects of rhGH and the possible progression of ventricular hypertrophy. However, earlier reports have shown that GH treatment is not associated with cardiac impairment in this group of patients [29].

In our NS patients, who did not show any cardiac defect at the beginning of the therapy, we reported no severe adverse events during GH-treatment, in particular no cardiac adverse events (i.e. hypertrophic cardiomyopathy). However, we suggest that, before starting rhGH treatment, every child with NSGHD should undergo cardiological evaluation with echocardiography and then a regular cardiological follow-up during rhGH therapy.

Furthermore, patients with NS are predisposed to have a higher risk for leukaemia and certain solid tumours, especially if they carry PTPN11 mutations [30]. Therefore, a close follow-up of these subjects is necessary in order to determine whether rhGH increases the risk of neoplasia.

In conclusion, pre-pubertal NS children with GHD slightly increase their height and growth rate during the first five years of GH therapy, although the response to GH treatment is significantly lower than IGHD children. Furthermore, the therapy appears to be safe since no severe adverse effects were reported, at least during the first five years. However, a close follow-up of these patients is mandatory, especially for monitoring cardiac function, haematological abnormalities or bleeding disorders.

## Abbreviations

BMI: Body mass index
GH: Growth hormone
IGF: Insulin-like growth factor
IGHD: Idiopathic growth hormone deficiency
NS: Noonan syndrome
NSGHD: Noonan syndrome growth hormone deficiency
SD: Standard deviation
SDS: Standard deviation score
